# Exploring Toxins for Hunting SARS-CoV-2 Main Protease Inhibitors: Molecular Docking, Molecular Dynamics, Pharmacokinetic Properties, and Reactome Study

**DOI:** 10.3390/ph15020153

**Published:** 2022-01-27

**Authors:** Mahmoud A. A. Ibrahim, Alaa H. M. Abdelrahman, Laila A. Jaragh-Alhadad, Mohamed A. M. Atia, Othman R. Alzahrani, Muhammad Naeem Ahmed, Moustafa Sherief Moustafa, Mahmoud E. S. Soliman, Ahmed M. Shawky, Paul W. Paré, Mohamed-Elamir F. Hegazy, Peter A. Sidhom

**Affiliations:** 1 Computational Chemistry Laboratory, Chemistry Department, Faculty of Science, Minia University, Minia 61519, Egypt; a.abdelrahman@compchem.net; 2 Department of Chemistry, Faculty of Science, Kuwait University, Kuwait City 13060, Kuwait; mostafa_msm@hotmail.com; 3 Cardiovascular and Metabolic Sciences Department, Lerner Research Institute, Cleveland Clinic, Cleveland, OH 44195, USA; 4 Molecular Genetics and Genome Mapping Laboratory, Genome Mapping Department, Agricultural Genetic Engineering Research Institute (AGERI), Agricultural Research Center (ARC), Giza 12619, Egypt; matia@ageri.sci.eg; 5 Department of Biology, Faculty of Science, University of Tabuk, Tabuk 71491, Saudi Arabia; o-alzahrani@ut.edu.sa; 6 Department of Chemistry, The University of Azad Jammu and Kashmir, Muzaffarabad 13100, Pakistan; drnaeem@ajku.edu.pk; 7 Molecular Modelling and Drug Design Research Group, School of Health Sciences, University of KwaZulu-Natal, Westville, Durban 4000, South Africa; soliman@ukzn.ac.za; 8 Science and Technology Unit (STU), Umm Al-Qura University, Makkah 21955, Saudi Arabia; amesmail@uqu.edu.sa; 9 Department of Chemistry & Biochemistry, Texas Tech University, Lubbock, TX 79409, USA; paul.pare@ttu.edu; 10 Chemistry of Medicinal Plants Department, National Research Centre, 33 El-Bohouth St., Dokki, Giza 12622, Egypt; elamir77@live.com; 11 Department of Pharmaceutical Chemistry, Faculty of Pharmacy, Tanta University, Tanta 31527, Egypt; peter.ayoub@pharm.tanta.edu.eg

**Keywords:** toxins, SARS-CoV-2 M^pro^, in silico screening, molecular docking calculations, molecular dynamics (MD) simulations, reactome

## Abstract

The main protease (M^pro^) is a potential druggable target in SARS-CoV-2 replication. Herein, an in silico study was conducted to mine for M^pro^ inhibitors from toxin sources. A toxin and toxin-target database (T3DB) was virtually screened for inhibitor activity towards the M^pro^ enzyme utilizing molecular docking calculations. Promising toxins were subsequently characterized using a combination of molecular dynamics (MD) simulations and molecular mechanics-generalized Born surface area (MM-GBSA) binding energy estimations. According to the MM-GBSA binding energies over 200 ns MD simulations, three toxins—namely philanthotoxin (T3D2489), azaspiracid (T3D2672), and taziprinone (T3D2378)—demonstrated higher binding affinities against SARS-CoV-2 M^pro^ than the co-crystalized inhibitor XF7 with MM-GBSA binding energies of −58.9, −55.9, −50.1, and −43.7 kcal/mol, respectively. The molecular network analyses showed that philanthotoxin provides a ligand lead using the STRING database, which includes the biochemical top 20 signaling genes CTSB, CTSL, and CTSK. Ultimately, pathway enrichment analysis (PEA) and Reactome mining results revealed that philanthotoxin could prevent severe lung injury in COVID-19 patients through the remodeling of interleukins (IL-4 and IL-13) and the matrix metalloproteinases (MMPs). These findings have identified that philanthotoxin—a venom of the Egyptian solitary wasp—holds promise as a potential M^pro^ inhibitor and warrants further in vitro/in vivo validation.

## 1. Introduction

The causative factor in COVID-19 infection is Severe Acute Respiratory Syndrome Coronavirus-2 (SARS-CoV-2), a novel *β*-coronavirus of the positive-stranded RNA virus that results in gastrointestinal, respiratory, and neurological symptoms in humans [1,2]. From December 2019, a gigantic economic epidemic has been disseminated globally because of COVID-19 disease [3,4]. As of 29 December 2021, more than 281 million confirmed cases and over 5.4 million international deaths had been reported [5]. A small number of vaccines have currently been approved under emergency use authorization [6]. Notwithstanding the weak vaccination rate, the deficiency of specific therapies, and the development of numerous viral variants, the pandemic goes on to distribute quickly and intricately. As a consequence, more outstanding efforts are required to discover safe and potent drugs against SARS-CoV-2.

SARS-CoV-2 main protease (M^pro^) is a crucial enzyme for viral gene replication, expression, and transcription [7,8,9]. Therefore, inhibition of the viral M^pro^ enzyme is a putative strategy towards SARS-CoV-2 antiviral drug development. Several in silico and experimental attempts have been made to repurpose approved drugs as prospective curative candidates for the remediation of COVID-19 [10,11,12,13]. Further, a combination of virtual screening and molecular dynamics (MD) simulations of chemical libraries towards SARS-CoV-2 targets has been executed [14,15,16,17]. Sundry small compounds have been identified during and after the first and second coronavirus prevalence waves. Among these, many edible plant-derived natural products and their related synthetic derivatives have attracted considerable interest as prospective COVID-19 drug candidates. Furthermore, natural products from marine species have recently demonstrated substantial antiviral characteristics [18,19,20,21]. Very recently, an emergency use authorization of PAXLOVID (PF-07321332), a covalent M^pro^ inhibitor with submicromolar activity developed by Pfizer, has been granted by the U.S. Food and Drug Administration (FDA) for treatment of patients with mild-to-moderate COVID-19 [22].

Toxin or toxin subunits have been used as therapeutic agents to treat an enormous number of diseases when they are not capable of causing damage or death to humanity [10,23]. Among the approved drugs, eleven drugs were recognized as toxins, such as exanta, ziconotide, exenatide, and lixisenatide [24]. Toxin and Toxin-Target Database (T3DB) database is an unparalleled bioinformatics resource that collects overall information about popular or omnipresent toxins and their toxin-targets into a single electronic storehouse [25]. The database includes more than 2900 small compounds and peptide toxins, over 33,000 toxin-target associations, and 1300 toxin-targets [25].

Exploring toxins to hunt potential inhibitors towards SARS-CoV-2 M^pro^ has not been conducted. So, in the current study, the T3DB database was virtually screened as M^pro^ specific drug candidates. Based on the predicted docking scores, the most potent toxins were submitted to molecular dynamics (MD) simulations combined with binding energy calculations using the molecular mechanics-generalized Born surface area (MM-GBSA) approach. Pathway enrichment analysis (PEA) and Reactome mining was performed to dissect biological aspects of the inhibitor hits on drug-target interactions with an interactive layout [16,26]. Such in silico screening can provide worthy insights with respect to the appropriateness of the obtained hits as future development of prospective clinical candidates.

## 2. Results and Discussion

In searching for small compounds to prohibit viral replication and transcription, in silico techniques were utilized to explore a chemical library containing more than 3678 toxins as potential SARS-CoV-2 M^pro^ inhibitors. The employed protocol was first validated based on available experimental data.

### 2.1. Validation of In Silico Protocol

**Figure 1:**
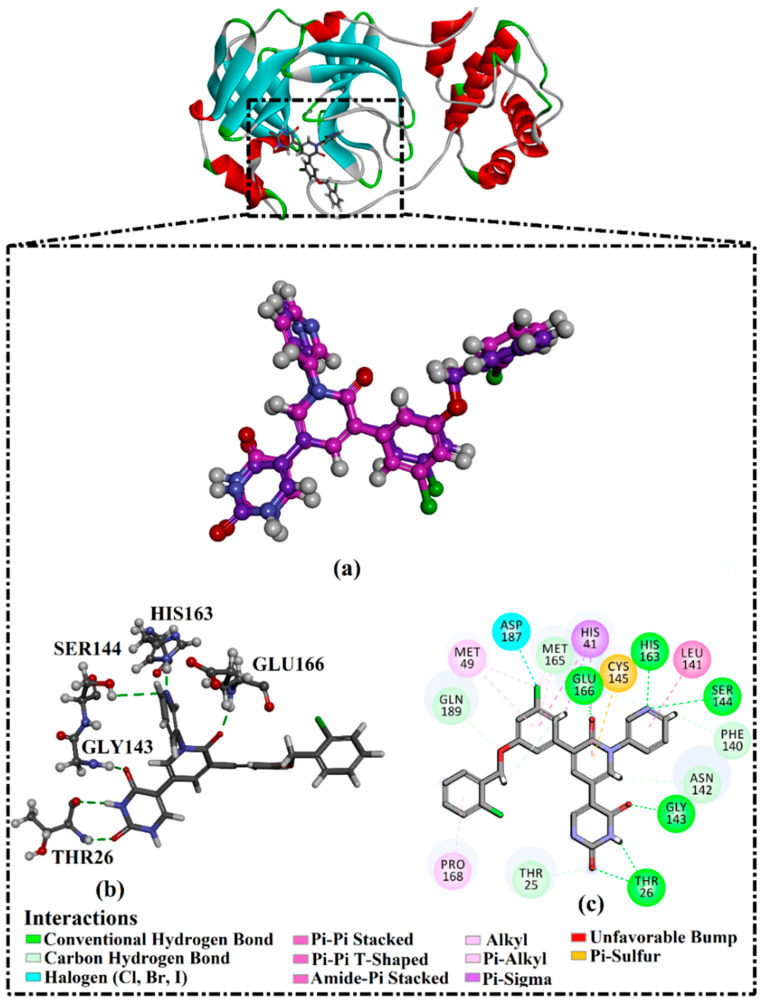
(**a**) 3D representation of the anticipated docking pose of XF7 (in pink) and experimental structure (in mauve) of XF7, (**b**) 3D, in addition to (**c**) 2D representations of the predicted binding mode of XF7 complexed with SARS-CoV-2 main protease (M^pro^).

The performance of the utilized in silico protocol to predict the binding mode of SARS-CoV-2 M^pro^ inhibitor was evaluated. The co-crystallized (5*S*)-5-(3-{3-chloro-5-[(2-chlorophenyl)methoxy]phenyl2-oxo[2*H*-[1,3′-bipyridine]]-5-yl)pyrimidine-2,4(3*H*,5*H*)-dione (XF7) inhibitor was redocked against the SARS-CoV-2 M^pro^, as well as the anticipated binding mode was compared to the experimental binding mode (PDB code: 7L13) [27]. As shown in Figure 1, the predicted binding mode was very similar to the resolved experimental binding mode with an RMSD of 0.20 Å and a binding affinity of −9.5 kcal/mol. This data comparison revealed the superior performance of AutoDock4.2.6 software in anticipating the experimental binding mode of M^pro^ inhibitors. The robust binding of XF7 with M^pro^ is attributed to the NH and two CO groups of pyrimidine-2,4(1*H*,3*H*)-dione ring to form hydrogen bonds with a backbone CO, NH group of THR26 and the backbone NH of GLY143 with bond lengths of 2.49, 2.28, and 2.07 Å, respectively (Figure 1). Besides, a nitrogen atom of pyridine rings interacts with the backbone CO group of SER144 and the imidazole ring of HIS163 with bond lengths of 3.29 and 1.91 Å, respectively (Figure 1). At the same time, the carbonyl group of pyridin-2(1*H*)-one ring exhibits a hydrogen bond with the backbone NH group of GLU166 with a bond length of 1.81 Å (Figure 1). Therefore, the docking protocol confirms the outperformance of this approach in identifying potent inhibitors as prospective SARS-CoV-2 M^pro^ inhibitors.

### 2.2. T3DB Database Virtual Screening

To identify M^pro^ inhibitors from toxins, AutoDock4.2.6 software was employed to virtually screen the T3DB database. At the outset, the T3DB database was filtered towards M^pro^ with conventional docking parameters. According to the portended binding affinity, 200 toxins demonstrated docking scores less than −8.0 kcal/mol towards M^pro^. Therefore, those 200 toxins were submitted to more elaborate molecular docking calculations with costly docking parameters. The evaluated docking scores for the top 200 hits are summarized in Table S1. Thirty-two toxins displayed docking scores less than the co-crystalized ligand (XF7 = −9.5 kcal/mol). 2D docking poses showing key amino acids inside the M^pro^’s binding site are illustrated in Figure S1. Most of the scrutinized toxins demonstrated similar binding modes inside the M^pro^’s active site, forming a fundamental hydrogen bond with GLN189 and GLU166 (Figure S1). 2D chemical structures and calculated docking scores for those toxins are listed in Table 1.

**Figure 2:**
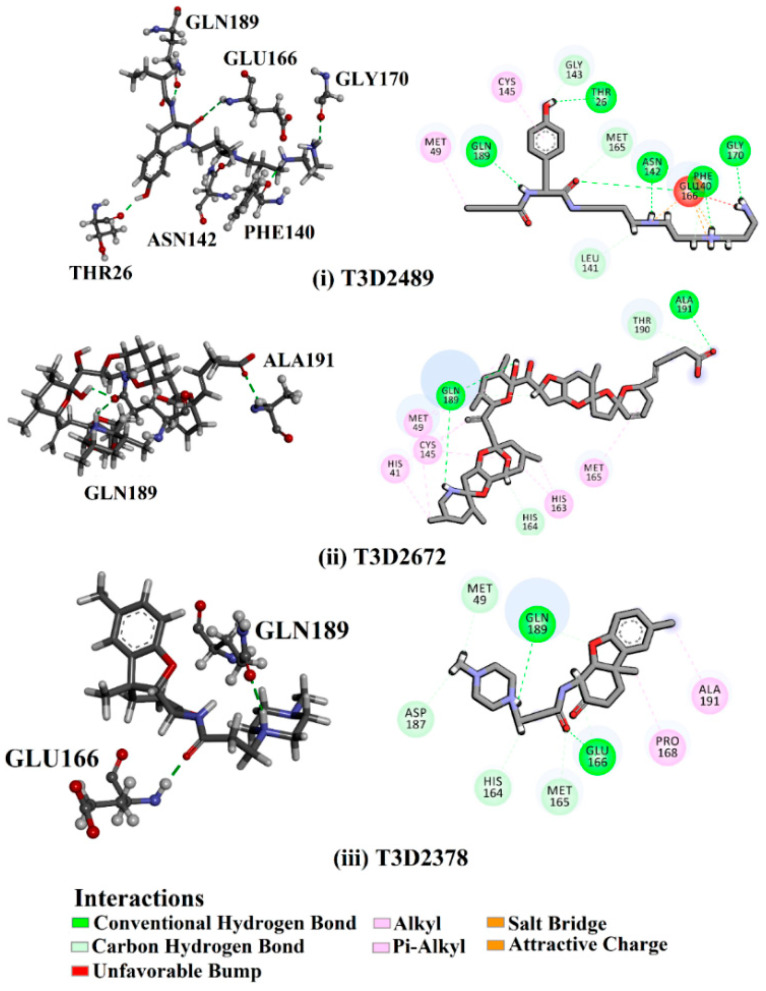
3D and 2D molecular interactions of the predicted docking poses of toxins (**i**) T3D2489, (**ii**) T3D2672, and (**iii**) T3D2378 towards SARS-CoV-2 M^pro^.

Philanthotoxin (T3D2489), a component of the venom of the Egyptian solitary wasp, demonstrated the highest binding affinity with a docking score of −11.7 kcal/mol, displaying a total of six hydrogen bonds with the key amino acid residues of M^pro^ (Table 1). Inspecting its binding mode showed that the ammonium group (NH_3_^+^) participates in a hydrogen bond with the backbone CO group of GLY170 with a bond length of 2.25 Å (Figure 2). Furthermore, two dimethylaminium groups participate in two hydrogen bonds with the backbone CO groups of PHE140 and ASN142 with bond lengths of 1.98 and 1.75 Å, respectively (Figure 2). The CO and NH of two N-methylacetamide groups display two hydrogen bonds with the backbone NH of GLU166 and the backbone carbonyl of GLN189 with bond lengths of 2.97 and 2.19 Å, respectively (Figure 2). The hydroxy group of the phenol ring interacts with the backbone NH of THR26 with a bond length of 1.99 Å (Figure 2). It is worth mentioning that gram-scale synthesis of philanthotoxin analogs has been obtained [28].

Azaspiracid (T3D2672), an alkaloid from *Mytilus edulis* (blue mussel) [29], exhibited the second-greatest binding affinity towards M^pro^ with a docking score of −11.6 kcal/mol (Table 1). Inspecting the binding mode of T3D2672 within the M^pro^’s binding pocket disclosed that the OH group of the tetrahydro-3,5-dimethyl-2*H*-pyran-2-ol ring and NH of 3,5-dimethylpiperidine ring exhibit two hydrogen bonds with the backbone CO group of GLN189 with bond lengths of 2.1 and 2.18 Å, respectively (Figure 2). Furthermore, CO of the carboxylate group contributes a hydrogen bond with the backbone NH of ALA191 with a bond length of 2.23 Å (Figure 2).

Taziprinone (T3D2378), a *β*-amino acid derivative, also showed a strong binding affinity against M^pro^ with an average value of −11.2 kcal/mol. The interaction was based in part on two hydrogen bonds with GLN189 and GLU166 with bond lengths of 2.32 and 1.85 Å, respectively (Figure 2).

### 2.3. Molecular Dynamics (MD) Simulations

**Figure 3:**
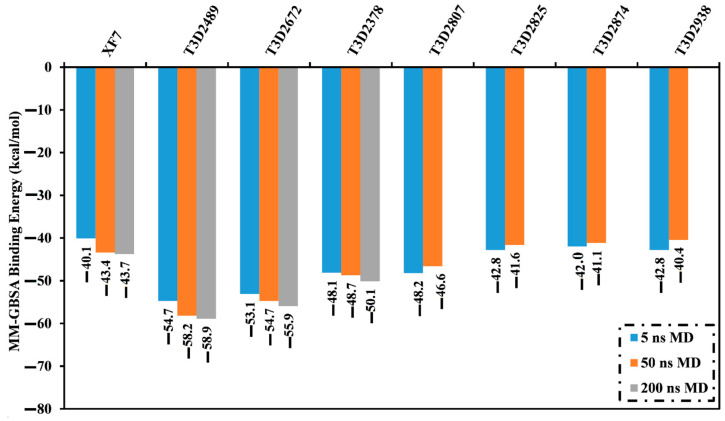
Calculated MM-GBSA binding energies for the native XF7 inhibitor and the most seven potent toxins complexed with SARS-CoV-2 M^pro^ throughout 5 ns, 50 ns, and 200 ns MD simulations.

Molecular dynamics (MD) simulations investigate the stabilization of the inhibitor-receptor complexes, conformational pliabilities, the reliability of inhibitor-receptor binding energies, and structural details [30]. Therefore, the most potent toxins with docking scores lower than −9.5 kcal/mol were subjected to MD simulations, followed by binding affinity estimations. The simulations were executed for 5 ns to reduce time and computational costs. The corresponding MM-GBSA binding affinities were computed and summarized in Table S2. From these data, it is apparent that seven toxins showed lower binding energies (Δ*G*_binding_) compared to the co-crystallized XF7 inhibitor (calc. −40.1 kcal/mol) (Figure 3). As a result, these toxins were selected and subjected to a 50 ns MD simulation to obtain more meticulous M^pro^ binding affinities. In addition, MM-GBSA binding energies were evaluated (Figure 3). Three out of these seven toxins—namely T3D2489, T3D2672, and T3D2378—displayed lower binding energies (Δ*G*_binding_) than the co-crystallized XF7 inhibitor (calc. −43.4 kcal/mol). The evaluated MM-GBSA binding affinities for T3D2489, T3D2672, and T3D2378 towards M^pro^ were −58.2, −54.7, and −48.7 kcal/mol over 50 ns MD simulations, respectively. To obtain more reliable binding affinities, longer MD simulations of 200 ns were executed for those three potent toxins in complex with M^pro^, and the corresponding binding affinities were computed (Figure 3).

There is no noteworthy disparity between the computed MM-GBSA binding affinities throughout 50 ns and 200 ns MD simulations for T3D2489-, T3D2672-, and T3D2378-M^pro^ complexes (Figure 3). Compared to the binding energy of the co-crystallized XF7 (calc. −43.7 kcal/mol), T3D2489, T3D2672, and T3D2378 revealed a greater binding affinity against M^pro^ throughout the 200 ns MD simulations with an average Δ*G*_binding_ of −58.9, −55.9, and −50.1 kcal/mol, respectively.

**Figure 4:**
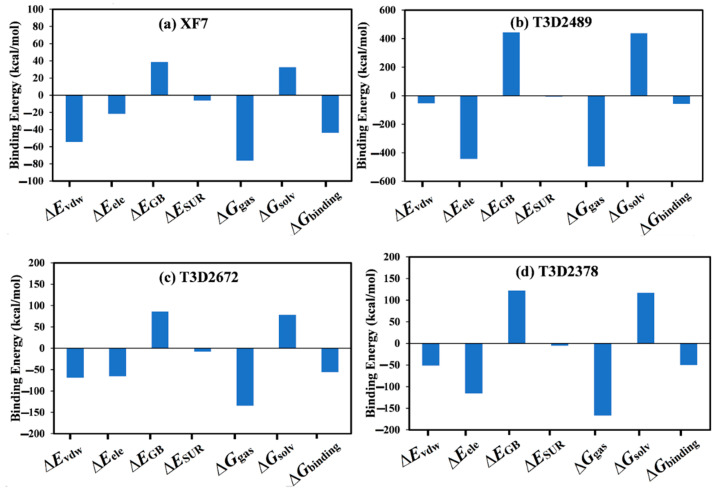
Components of the MM-GBSA binding energies for (**a**) XF7, (**b**) T3D2489, (**c**) T3D2672, and (**d**) T3D2378 complexed with SARS-CoV-2 M^pro^ throughout the simulation time of 200 ns.

To identify the principal driving forces in binding the identified toxins with M^pro^, decomposition of the MM-GBSA binding affinities was carried out (Figure 4). *E*_vdw_ was a significant contributor in the XF7-M^pro^ binding affinity with an average value of −54.5 kcal/mol; besides, *E*_ele_ contribution was favorable with an average value of −21.7 kcal/mol (Figure 4). For compounds T3D2489 and T3D2378, a predominance of *E*_ele_ forces was observed with a value of −442.9 and −115.8 kcal/mol, respectively (Figure 4). *E*_vdw_ was also favorable, with an average value of −53.3 and −51.2 kcal/mol for the T3D2489- and T3D2378-M^pro^ complexes, respectively (Figure 4). On the other hand, *E*_vdw_ and *E*_ele_ had approximately the same contribution in T3D2672-M^pro^ binding affinity with average values of −68.9 and −65.4 kcal/mol (Figure 4).

**Figure 5:**
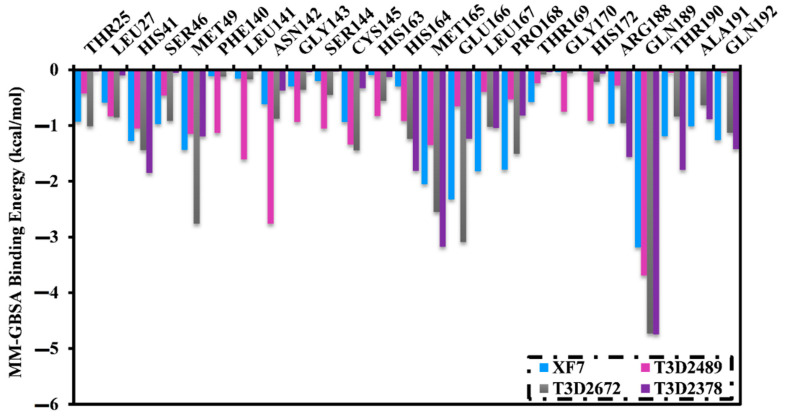
Energy participation of the proximal residues to the total binding free energy (kcal/mol) of T3D2489, T3D2672, T3D2378, and XF7 complexed with SARS-CoV-2 main protease (M^pro^).

To more fully scrutinize enzyme-inhibitor interactions and the participation of proximal amino acids in the inhibitor-enzyme complexes, total Δ*G*_binding_ values were separated to individual residues with the assistance of an MM-GBSA approach (Figure 5); only residues with Δ*G*_binding_ values lower than −0.50 kcal/mol were considered. GLY143, HIS164, GLU166, and GLN189 interact with T3D2489, T3D2672, T3D2378, and XF7. GLN189 contributed to the total binding affinity significantly with values of −3.7, −4.7, −4.7, and −3.2 kcal/mol for T3D2489-, T3D2672-, T3D2378-, and XF7-M^pro^ complexes, respectively (Figure 5). GLU166 was the second-highest contributor to total binding free with values of −0.7, −3.1, −1.2, and −2.3 kcal/mol for T3D2489-, T3D2672-, T3D2378-, and XF7-M^pro^ complexes, respectively (Figure 5). It is worth mentioning that all investigated complexes have similar interaction patterns with key amino acid residues, which signifies a resemblance in the binding mode in these complexes.

### 2.4. Post-MD Analyses

To further examine the constancy of T3D2489, T3D2672, and T3D2378 complexed with M^pro^, structural and energetical analyses were executed over 200 ns MD simulations and compared to those of the co-crystalized XF7 inhibitor. Monitoring the structural steadiness of the scrutinized complexes was effectuated via inspecting hydrogen bond length, root-mean-square deviation (RMSD), binding energy per frame, and center-of-mass (CoM) distance.

#### 2.4.1. Binding Energy per Frame

**Figure 6:**
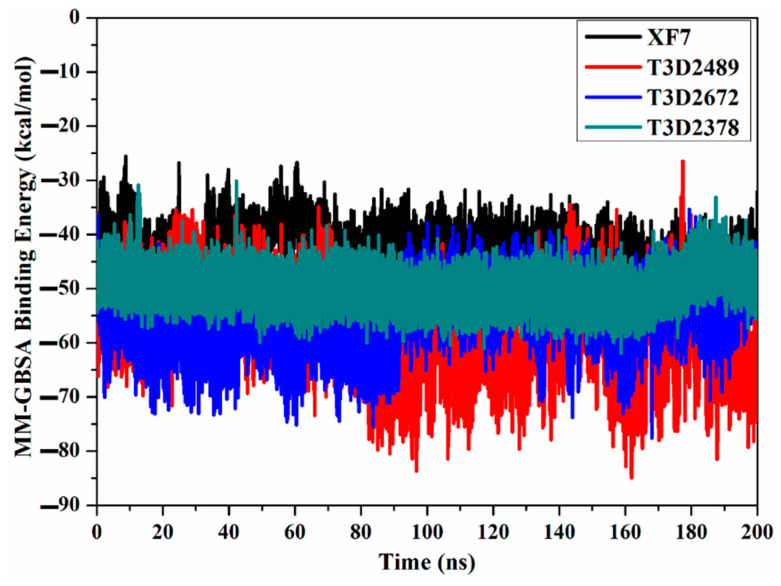
Computed MM-GBSA binding energy per frame for XF7 (in black), T3D2489 (in red), T3D2672 (in blue), in addition to T3D2378 (in cyan) in complex with SARS-CoV-2 main protease (M^pro^) throughout 200 ns MD simulations.

The structural steadiness of T3D2489, T3D2672, T3D2378, and XF7 complexed with M^pro^ was comprehensively evaluated over the 200 ns MD simulations via mensuration correlations between binding affinity and time (Figure 6). An interesting aspect of binding energy per frame was the overall stabilities for T3D2489, T3D2672, T3D2378, and XF7 with average Δ*G*_binding_ of −58.9, −55.9, −50.1, and −43.7 kcal/mol, respectively. Based on this analysis, all investigated systems preserved constancy over the 200 ns MD simulations.

#### 2.4.2. Intermolecular Hydrogen Bonds

**Figure 7:**
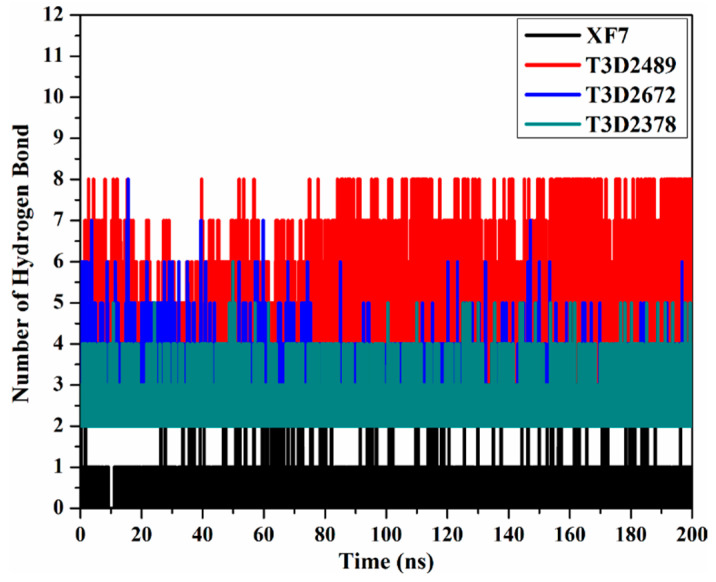
Number of hydrogen bonds formed between XF7 (in black), T3D2489 (in red), T3D2672 (in blue), and T3D2378 (in cyan) and SARS-CoV-2 M^pro^ throughout 200 ns MD simulations.

Hydrogen bond analysis was used to estimate the constancy of hydrogen bonding between identified toxins and M^pro^ throughout a 200 ns MD simulation. The number of hydrogen bonds per frame was evaluated and depicted in Figure 7. The number of hydrogen bonds oscillated throughout the 200 ns MD simulations, and the average number of hydrogen bonds was four, two, and two for T3D2489-, T3D2672-, and T3D2378-M^pro^ complexes. It is worth noting that XF7 showed the lowest number of hydrogen bonds with the proximal amino acids within M^pro^’s binding pocket, while the outstanding binding affinity of XF7 with average Δ*G*_binding_ of −43.7 kcal/mol may be ascribed to other interactions like van der Waals and hydrophobic interactions. The dominance of van der Waals feature of interactions of XF7 with M^pro^ is in agreement with the MM-GBSA binding energies decomposition (Figure 4). The results obtained from the intermolecular bonds assured the presentence of a great stationary for the T3D2489, T3D2672, and T3D2378 complexed with M^pro^ compared to the XF7-M^pro^ complex.

#### 2.4.3. Center-of-Mass Distance

**Figure 8:**
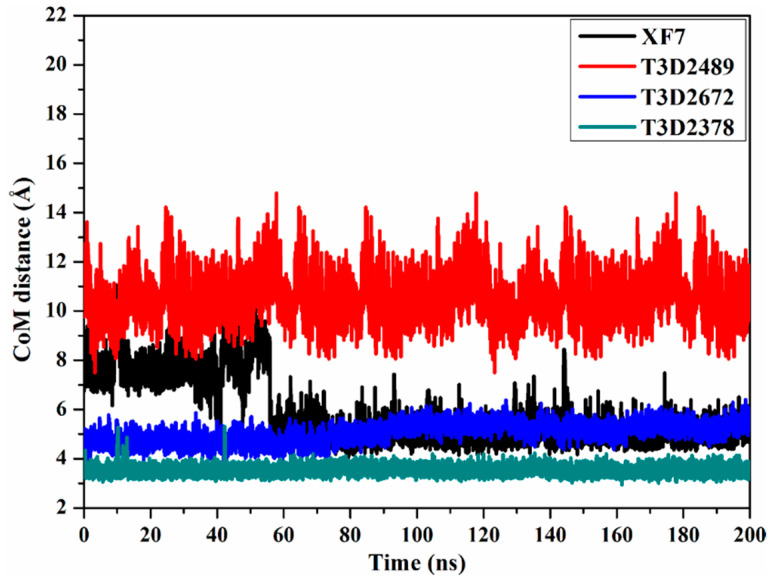
Distance between the center-of-mass (CoM) (in Å) of XF7 (in black), T3D2489 (in red), T3D2672 (in blue), and T3D2378 (in cyan) and GLN189 of SARS-CoV-2 M^pro^ over the 200 ns MD simulations.

To obtain further in-depth insight into the steadiness of toxin-M^pro^ complexes throughout the 200 ns MD simulations, the center-of-mass (CoM) distance was estimated between the toxin and GLN189 (Figure 8). From the CoM graph, it is apparent that the measured CoM distance was more stable for T3D2672 and T3D2378 in complex with M^pro^ than T3D2489 and XF7 with average values of 5.1, 3.5, 10.2, and 5.6 Å, respectively. These findings demonstrate that the most identified toxins bind more tightly with the SARS-CoV-2 M^pro^ than XF7.

#### 2.4.4. Root-Mean-Square Deviation

**Figure 9:**
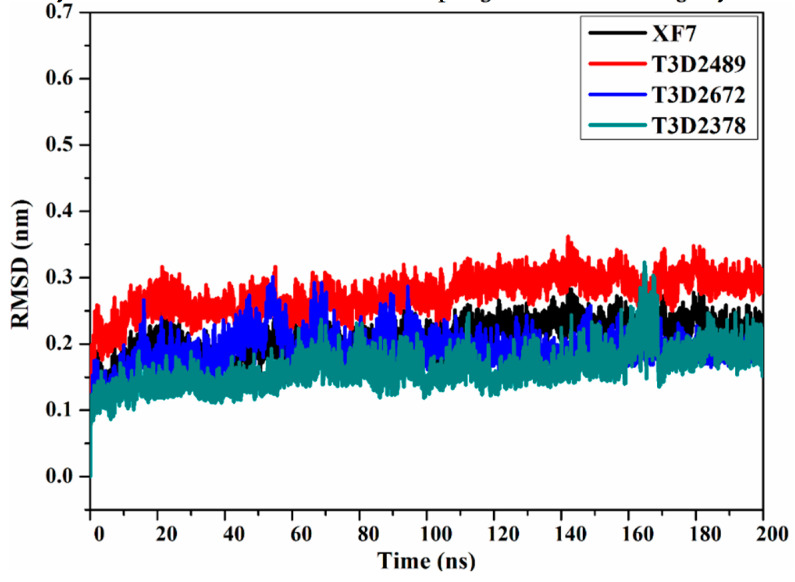
Root-mean-square deviation (RMSD) of the backbone atoms from the initial structure of XF7 (in black), T3D2489 (in red), T3D2672 (in blue), and T3D2378 (in cyan) with SARS-CoV-2 M^pro^ during the simulation time of 200 ns.

The principal objective of the MD simulations is to inspect the positional and conformational changes of ligands upon binding to the binding pocket, which supplies insight into the binding steadiness. For ease of comparison, the root-mean-square deviation (RMSD) of the entire complex backbone atoms were evaluated to examine the structural stability of the T3D2489, T3D2672, T3D2378, and XF7 in complex with M^pro^ (Figure 9). Unambiguously, the evaluated RMSD values for the identified toxins complexed with M^pro^ stayed below 0.25 nm throughout the MD simulations (Figure 9). It is worth noting that RMSD analysis shows that the complexes stabilized after 10 ns and conserved their stabilities up to the end of the simulations. In general, the current findings confirmed that T3D2489, T3D2672, and T3D2378 are tightly bonded and do not leverage the structural constancy of the M^pro^, in addition to keeping structural integrity.

### 2.5. Drug-like Properties

The efficacy of therapeutic medicaments is substantially based on the molecular characteristics and bioactivity of the chemical compounds [31]. To predict the drug-like and bioactivity of the identified toxins as SARS-CoV-2 inhibitors, a Molinspiration tool was employed to estimate the in silico molecular characteristics. The anticipated physiochemical properties are summarized in Table 2. Promising drug-likeness properties were observed, except for T3D2672, which showed violations in some parameters such as _mi_logP, TPSA, number of hydrogen bonds acceptors (nON), and molecular weight. The _mi_logP values of T3D2489, T3D2378, and XF7 were promising, with values less than 5 [32]. The TPSA values of the T3D2489, T3D2378, and XF7 were less than 140 Å, revealing that the compounds have eminent oral absorption or membrane permeability [33]. In addition, the number of hydrogen bond acceptors (nON) was lower than 10, while the number of hydrogen bond donors (nOHNH) was lower than 5, except for T3D2489. The molecular weights for T3D2489, T3D2378, and XF7 were 435.6, 385.5, and 492.5 daltons, respectively, proposing that most investigated toxins have good absorption and/or permeation across the cell membrane.

### 2.6. In Silico ADMET Analysis

ADMET analysis is especially helpful in simplifying clinical trials, especially in the early stage of drug design [34]. GI absorption, skin, Caco2 permeability, and aqueous solubility are absorption properties needed to be considered in any drug discovery process [35]. It is signified that a GI absorption value greater than 30% indicates perfect absorbance. T3D2489 (47.6%), T3D2672 (56.7%), T3D2378 (95.0%), and XF7 (93.5%) manifested good absorbance rates (Table 3). The identified toxins revealed appropriate skin permeability, with a skin permeability value higher than −2.5 cm/h. The identified toxins also showed low Caco2 permeability (<0.9 cm/s). Another substantial agent through ADMET analysis was to anticipate the P-glycoprotein non-substrate. All toxins were identified as a substrate for P-glycoprotein (Table 3).

In order to examine the drug distribution, the VDss, BBB membrane permeability, and CNS were assessed [32]. Higher distribution volumes were observed for T3D2489, T3D2672, and T3D2378 with log VDss values of 1.6, 0.6, and 1.3, respectively (Table 3). For BBB membrane permeability, log BB values in the range of −1.0 to 0.3 implied that the drug candidates passed the BBB membrane. For CNS permeability, log PS values ranged from −3 to −2, pointing out impenetrability. Most of the investigated inhibitors were forecasted to be neither able to permeate the CNS nor pass the BBB membrane (Table 3).

CYP450 has a substantial role in drug metabolism in the liver system [36]. The metabolism scores revealed that all the inspected inhibitors did not prohibit CYP2D6 enzymes and did not perform as inhibitors for CYP3A4, CYP2C9, and CYP2C19 enzymes, except for the reference compound. The total drug clearance was inspected via a collection of hepatic and renal clearance. Total clearance construes the drug concentration in the body utilizing its removal rate. The data indicated that compound excretion rates range from −0.1 to 1.4 mL/min/kg (Table 3).

In drug design, toxicity is a significant criterion and plays a remarkable role in selecting of sufficient drug candidates [32]. All the drug candidates in this analysis have not passed any skin allergic action and hepatotoxic influence (Table 3). hERG inhibition (I and II) is a fundamental agent for toxicity analysis in addition to it also including cardiotoxicity. None of the inhibitors displayed inhibitory behaviors for hERG I. T3D2489, T3D2378, and XF7 were foretold to be hERG II inhibitors. All the investigated inhibitors had not crossed any *Tetrahymena pyriformis* as well as AMES toxicities. The maximum tolerated dosage range, lowest-observed-adverse-effect level (LOAEL), and LD_50_ were expected via the toxicity analysis server and the anticipated scores as summarized in Table 3. Consequently, this study concluded that these bioactive identified toxins could be utilized as possible M^pro^ inhibitor candidates.

### 2.7. Molecular Target Prediction and Network Analysis

**Figure 10:**
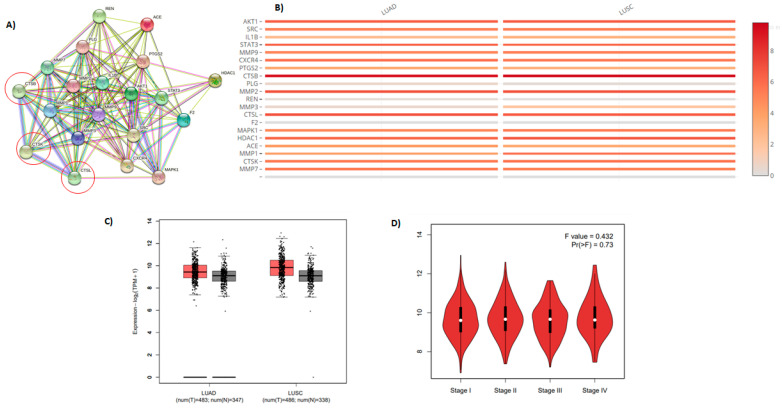
Bioinformatic analysis: (**A**) The PPI network of top genes; (**B**) the top 20 gene comparison in lung adenocarcinoma and lung squamous cell carcinoma; (**C**,**D**) Gene Expression Profiling Interactive Analysis (GEPIA), the expression level of CTSB was increased in lung adenocarcinoma and squamous cell lung carcinoma.

The lysosomal cathepsins, primarily cathepsin L (CTSL) and cathepsin B (CTSB) cleave and activate S proteins, which then merge with host cells [37,38]. SARS-CoV-2 upregulates CTSL expression both in vivo and in vitro [39]. This increases pseudo-virus infection in human cells. CTSL may be a therapeutic target to remedy COVID-19 disease [40,41]. CatL is a secreted lysosomal protease that is also known as a lysosomal protease. Excessive production of cysteine cathepsins has been related to various clinical conditions, including inflammation. As a result, high quantities of CatL might be activated in inflammatory cells under inflammatory circumstances. Moreover, because this enzyme is involved in the genesis, progression, and metastasis of cancer, its inhibition might be beneficial in treatment [42]. The plasma CatL has been recommended as a marker for pancreatic cancer. Philanthotoxin (T3D2489) was identified as an inhibitor against M^pro^ and provided a ligand lead using the STRING database, including the biochemical top 20 signaling genes CTSB, CTSL, and CTSK (Figure 10 and Table S3).

### 2.8. Pathway Enrichment Analysis (PEA) and Reactome Mining

**Figure 11:**
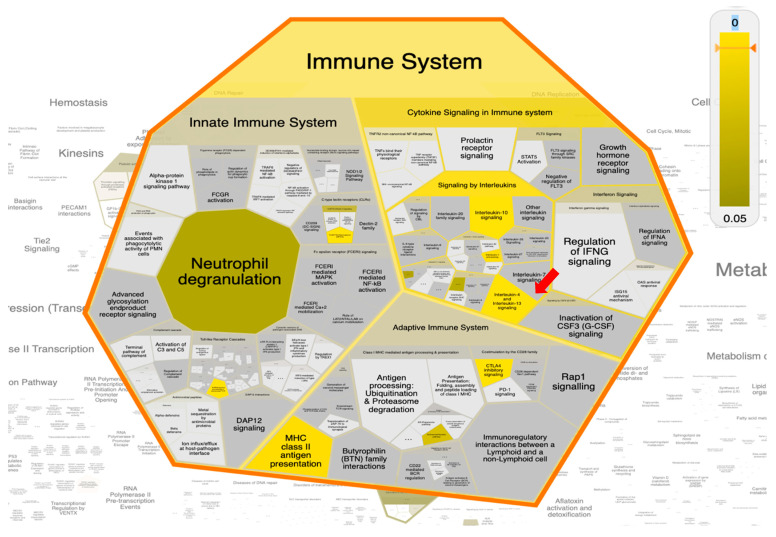
The Reacfoam map shows the top enriched pathway (Interleukin-4 and Interleukin-13 signaling) influenced by the top 20 gene targets in response to philanthotoxin (T3D2489).

**Figure 12:**
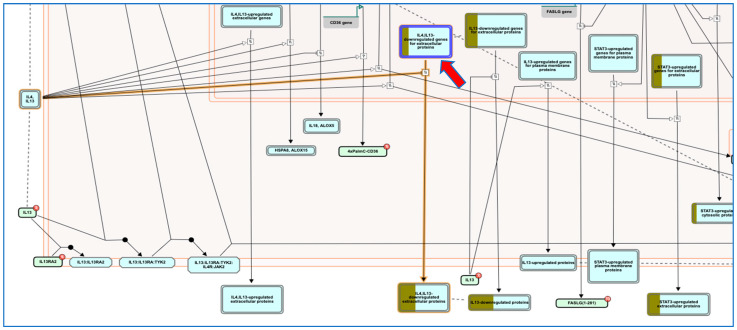
Graphic representation of the Interleukin-4 and Interleukin-13 signaling Reactome pathway influenced as a response to philanthotoxin (T3D2489) in the human genome.

For extensive and biological-wide mining of philanthotoxin (T3D2489) target-function interactions, a Reacfoam map was constructed based on PEA analysis and Reactome mining/modeling. The Reacfoam map tree was constructed to illustrate the top enriched pathways affected by 20 top gene targets in response to philanthotoxin (Figure 11). Moreover, an illustrative graphical map was built to show the top enriched pathway as a response to T3D2489 treatment (Figure 12). Prominently, the top enriched pathways involved: (1) signaling by the interleukin pathway, (2) interleukin-4 and interleukin-13 signaling pathway, and (3) immune system pathway. These pathways were determined to be the most significantly enriched pathways targeted by T3D2489 (Table S3).

The SARS-CoV-2 infection causes an induced inflammation and generates subsequent higher levels of immune cell infiltration and cytokines that trigger matrix metalloproteinases (MMPs) activation. Interestingly, the PEA-Reactome mining coupled approach revealed higher enrichment of signaling by Interleukins (particularly: Interleukin-4 and Interleukin-13 signaling), activation of matrix metalloproteinases signaling pathway, and immune system as a result of philanthotoxin induction among all other human biological pathways. Interestingly, remodeling of interleukins (IL-4 and IL-13) can transform growth factor-beta (TGF-*β*) levels, and consequently, the number of M2 macrophages in SARS-CoV-2 patients, which ultimately can prevent severe lung injury [43]. Furthermore, a recent report emphasized that gathering and remodeling the immune cells, matrix metalloproteinases, secreted cytokines (especially interleukin-4 and Interleukin-13), and several other mediators is proposed as a feasible option for treating COVID-patients [44].

## 3. Materials and Methods

### 3.1. Target Preparation

The X-ray resolved three-dimensional structure of SARS-CoV-2 main protease (M^pro^) (PDB ID: 7L13, resolution: 2.17 Å) in complex with a noncovalent inhibitor (XF7) was retrieved and utilized as a template for all in silico calculations [27]. The viral target was prepared by eradicating all heteroatoms involving ligands, water molecules, and ions. The protonation states of the titratable amino acids were investigated and assigned using the H++ web server [45]. In addition, all missing hydrogen atoms are topologically added. In H++ estimations, physiologic conditions of 10, 0.15, 80, and 6.5 for internal dielectric constant, salinity, external dielectric constant, and PH, respectively.

### 3.2. Database Preparation

The Toxin and Toxin Target Database (T3DB) was downloaded and prepared for virtual screening [25]. All the molecules were retrieved in a 2D structural data format (SDF). Omega2 software was then utilized to construct the 3D chemical structures [46,47]. A conformational search was executed to generate all conformers with an energy window of 10 kcal/mol. The lowest energy conformer was then minimized using an MMFF94S force field available within SZYBKI software [48,49]. Tautomer and fixpka applications implemented inside QUACPAC software were applied to investigate the tautomer enumeration and the protonation state of the toxins [50]. The partial atomic charges of each compound within the T3DB database were determined using a Gasteiger-Marsili method [51]. Duplicated molecules with congruent international chemical identifier keys (InChIKey) were removed [52]. Prepared T3DB data files are available through www.compchem.net/ccdb. An illustrative diagram of the employed computational approaches for the screening process of the T3DB database is illustrated in Figure S2.

### 3.3. Molecular Docking

AutoDock4.2.6 software was utilized to conduct all molecular docking calculations [53]. On the basis of the AutoDock protocol [54], the MGL tools (version 1.5.7) were used to generate the pdbqt file for the SARS-CoV-2 M^pro^. For molecular docking calculations, the number of generations and population size were set to 27,000 and 300, respectively. The maximum number of energy evaluations (*eval*) and the number of genetic algorithms (*GA*) run variables were adjusted to 5,000,000, and 25,000,000, and 50, and 250 for conventional and expensive molecular docking calculations, respectively. The rest of the docking parameters were maintained at the default settings. The grid box size was set at 60 Å × 60 Å × 60 Å, which is capable of accommodating the entire M^pro^’s binding site. Grid map files with a spacing value of 0.375 Å were established utilizing AutoGrid 4.2.6. The grid was centered at the coordinates X = −13.069, Y = 9.740, and Z = 68.490. A genetic algorithm inside the AutoDock software was employed to evaluate the various inhibitor conformers. Conformations were clustered via the root-mean-square deviation (RMSD) tolerance of 1.0 Å and were sorted based on the docking score [55]. Besides, the lowest docking score within the largest cluster was considered for opting as the representative pose.

### 3.4. Molecular Dynamics Simulations

AMBER16 software was used to conduct all molecular dynamics (MD) simulations for the most potent toxins in complex with SARS-CoV-2 M^pro^ [56]. A general AMBER force field (GAFF2) [57] was employed to characterize the investigated toxins, whereas the AMBER force field of 14SB [58] was adopted for the characterization of the viral enzyme. The restrained electrostatic potential (RESP) fitting approach was used to estimate the atomic partial charge of the studied toxins at the HF/6-31G* level with the assistance of Gaussian09 software [59,60]. A solvated octahedron box of TIP3P water model with 12 Å distances between the box edge and atoms of the toxin-M^pro^ complexes was constructed. The sodium (Na^+^) and chloride (Cl^−^) counter-ions were inserted to neutralize all solvated systems, as well as to preserve the isosmotic condition (0.15 M NaCl). The prepared systems were initially minimized through combined steepest and conjugate gradient algorithms for 5000 steps to remove any steric clashes or inappropriate geometries. The minimized systems were thereafter gradually heated to 300 K over a brief interval of 50 ps with a weak constraint of 10 kcal mol^−1^ Å^−1^ on the amino acid residues. To guarantee a reasonable initial structure, an equilibration stage was executed over a total duration of 1000 ps under a constant number of particles, pressure (1 atm), and temperature (NPT) ensemble. Eventually, the production runs were conducted over simulation times of 5 ns, 50 ns, and 200 ns. Snapshots were recorded and saved each 10 ps for post-MD analyses. A cutoff distance of 12 Å was used for the non-bonded interactions. The Particle-Mesh Ewald (PME) algorithm was utilized to estimate the long-range electrostatic interactions [61]. The collision frequency (gamma_ln) 1.0 ps^−1^ was applied to maintain the temperature at 298 K [62]. The Berendsen barostat with a pressure relaxation time of 2 ps was utilized to keep pressure constant [63]. The SHAKE algorithm with 2 fs integration step was employed to restrict all bonds including hydrogen atoms [64]. All MD simulations were carried out utilizing the CUDA version of pmemd in AMBER 16 software. All molecular docking calculations, MD simulations, as well as quantum mechanics (QM) evaluations were performed on the CompChem GPU/CPU hybrid cluster (hpc.compchem.net). All molecular interactions were visualized via the Discovery Studio module of Biovia software [65].

### 3.5. MM-GBSA Binding Energy

A molecular mechanic generalized Born surface area (MM-GBSA) approach was adopted to calculate binding free energies of the investigated toxins in complex with SARS-CoV-2 M^pro^. The MM-GBSA (Δ*G*_binding_) binding energies were computed based on uncorrelated snapshots collected over the MD simulations as follows:

∆*G*_binding_ = *G*_complex_ − (*G*_toxin_ + *G*_Mpro_) (1)

where the energy term (*G*) is calculated as:

*G* = *E*_vdw_ + *E*_ele_ + *G*_GB_ + *G*_SA_ (2)

*E*_ele_ and *E*_vdw_ stand for electrostatic and van der Waals energies, respectively. *G*_SA_ implies the nonpolar solvation-free energy, generally computed with a linear relation to the solvent-accessible surface area (SASA). *G*_GB_ indicates the electrostatic solvation free energy estimated from the generalized Born equation. In this study, the modified GB model developed via Onufriev et al. (igb = 2) was used [66]. A single-trajectory method was utilized, in which the coordinates of every toxin-M^pro^, toxin, and M^pro^ were calculated from a single trajectory. Due to the expensive computational demand, entropy estimations were neglected [67,68].

### 3.6. Drug-Likeness Properties

For the most promising toxins, the physicochemical properties were estimated utilizing an online Molinspiration cheminformatics package (http://www.molinspiration.com) according to Lipinski Rules. Molinspiration supports for prediction of significant physicochemical properties such as molecular weight (MW), topological polar surface area (TPSA), octanol/water partition coefficient (_mi_logP), hydrogen bond donor (HBD), hydrogen bond acceptor (HBA), rotatable bond count (RB), and percent absorption (%ABS). %ABS was computed as follows [69]:

%ABS = 109 − [0.345 × TPSA](3)

### 3.7. In Silico ADMET Analysis

A freely accessible web server, pkCSM (http://biosig.unimelb.edu.au/pkcsm/prediction) is an in silico tool for anticipating substantial pharmacokinetic properties [34]. ADMET properties involve absorption (A): human intestinal absorption (HIA), water-solubility, P-glycoprotein I and II inhibitors, Caco-2 permeability, P-glycoprotein substrate, skin permeability. Distribution (D) is anticipated according to blood-brain barrier (BBB) permeability, steady-state volume of distribution (VDss), fraction unbound, and central nervous system (CNS) permeability. CYP2D6/CYP3A4 substrate is used to detect the metabolism (M). Excretion (E) is determined via drug total clearance. Subsequently, the toxicity (T) of the drugs is predicated on the Human Ether-a-go-go-related gene inhibition, carcinogenic status, mutagenic status, as well as acute oral toxicity [31].

### 3.8. Protein Interaction Network Analyses

To identify the probable targets for each ligand, the target molecules were tested using SwissTargetPrediction (http://www.swisstargetprediction.ch), an internet website-based program. We obtained the top one hundred genes for the possible metabolite. After that, a functional STRING database for the most likely targets was utilized to build protein-protein interactions (PPI). According to network architecture, Cytoscape 3.8.2 was utilized to analyze all possible receptor-function relationships. Pathway enrichment analysis was also performed utilizing Cytoscape 3.8.2 to scrutinize all prospective receptor-function connections for the top 20 targeted genes. The top 20 genes were investigated further using a newly developed interactive webserver (Gene Expression Profiling Interactive Analysis, GEPIA, http://gepia.cancer-pku.cn/index.html). Besides, to investigate all potential target-function relationships for the top most 20 targeted genes and their biological influenced-pathways/networks, the pathway enrichment analysis (PEA) approach was conducted with the assistance of Cytoscape 3.8.2 software [70]. Subsequently, the Reactome mining analysis and visualization were achieved using the ReactomeFIViz tool towards modeling and annotating all the philanthotoxin (T3D2489)-target interactions [71].

## 4. Conclusions

Herein, a toxin and toxin-target database (T3DB) was mined to identify potential SARS-CoV-2 M^pro^ inhibitors utilizing combined molecular docking and molecular dynamics simulations. On the basis of molecular docking calculations and MD simulations combined with molecular mechanics-generalized born surface area binding energy calculations, three compounds, T3D2489, T3D2672, and T3D2378, demonstrated promising binding affinity with Δ*G*_binding_ < −50.0 kcal/mol towards M^pro^. The energetic and structural analyses during 200 ns MD simulations pointed to great constancy for these compounds in complex with M^pro^. Additionally, the identified toxins demonstrated favorable pharmacokinetic and pharmacodynamic properties. The results obtained from PEA analysis combined Reactome-mining presented an interesting primary role of philanthotoxin in remodeling the interleukins (especially IL-4, IL -13) and matrix metalloproteinases (MMPs), which could alleviate lung injury in COVID-19 patients. In vitro and in vivo evaluations are planned to elucidate further the role of these compounds as prospective drug candidates and validate the computational findings.

## Figures and Tables

**Table 1:** Estimated conventional and expansive docking scores (in kcal/mol), 2D chemical structures, and origin/usage for most promising potent toxins towards SARS-CoV-2 M^pro a^.

Compound Name/Code	Origin/Usage ^b^	Chemical Structure	Docking Score (kcal/mol)		Compound Name/Code	Origin/Usage ^b^	Chemical Structure	Docking Score kcal/mol)	
			Conv. ^c^	Exp. ^d^				Conv. ^c^	Exp. ^d^
XF7		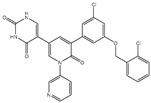	−9.1	−9.5	T3D2324	Industrial/workplace toxin	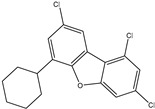	−9.2	−9.9
T3D2489	Insect toxin (Egyptian solitary wasp)	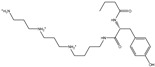	−11.7	−11.7	T3D2680	Synthetic compound (anticholesteremic agent)	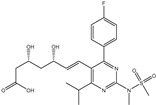	−9.8	−9.9
T3D2672	Marine toxin (Mytilus edulis)	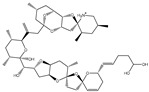	−11.3	−11.6	T3D2884	Synthetic compound (antineoplastic agent)	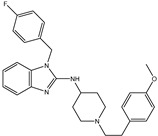	−9.8	−9.9
T3D2378	Industrial/workplace toxin	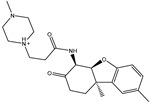	−11.2	−11.2	T3D4082	Plant toxin (*Veratrum californicum*)	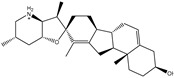	−9.8	−9.8
T3D2807	Synthetic compound (anti-anxiety agent)	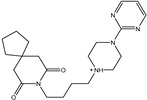	−10.9	−10.9	T3D2694	Food toxin (antihypoparathyroid agent)	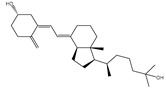	−9.7	−9.8
T3D2825	Synthetic compound (antihypertensive agent)	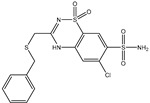	−10.9	−10.9	T3D2871	Food toxin (antipsychotic agent)	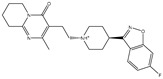	−9.7	−9.8
T3D2874	Synthetic compound (anti-allergic agent)	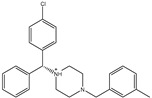	−10.8	−10.9	T3D4050	Plant toxin (*Solanum chacoense*)	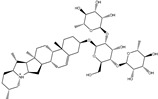	−9.6	−9.8
T3D2938	Synthetic compound (anti-allergic agent)	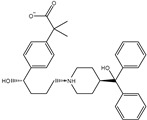	−10.5	−10.8	T3D2536	Animal toxin (*B. rubescens, B. marinus*)	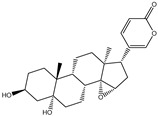	−9.6	−9.8
T3D2913	Synthetic compound (antipsychotic agent)	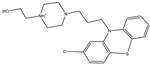	−10.5	−10.8	T3D2933	Synthetic compound (antipsychotic agent)	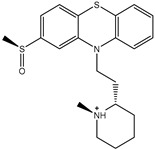	−9.6	−9.7
T3D4084	Plant toxin (genus *Veratrum*)	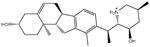	−10.2	−10.6	T3D4051	Marine toxin (*Tiostrea chilensis*)	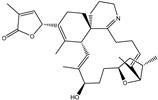	−9.5	−9.7
T3D2727	Synthetic compound (antineoplastic agent)	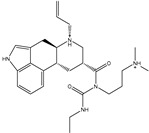	−10.1	−10.5	T3D2527	Animal toxin (genus *Dendrobates* and genus *Phyllobates*)	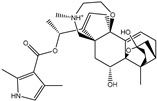	−9.5	−9.7
T3D2460	Synthetic compound (vasoconstrictor agent)	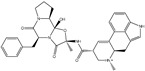	−10.1	−10.2	T3D0233	Synthetic compound (pesticide)	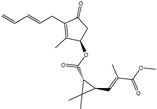	−9.5	−9.7
T3D2750	Synthetic compound (vasoconstrictor agent)	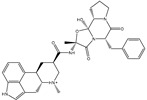	−10.0	−10.2	T3D2910	Synthetic compound (cholinesterase inhibitor)	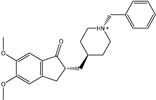	−9.5	−9.6
T3D2801	Synthetic compound (psychotropic drug)	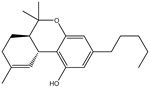	−10.0	−10.1	T3D2863	Synthetic compound (anti-HIV agent)	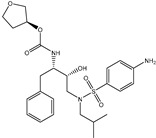	−9.5	−9.6
T3D4083	Plant toxin (genus *Veratrum*)	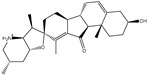	−9.9	−10.1	T3D2535	Animal toxin (*B. gargarizans*)	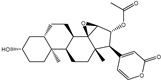	−9.4	−9.6
T3D2939	Synthetic compound (vasoconstrictor agent)	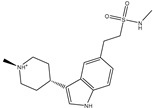	−9.8	−10.0	T3D4232	Bacterial toxin (cyanobacteria)	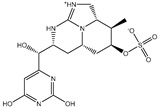	−9.3	−9. 6
T3D2143	Industrial/workplace toxin	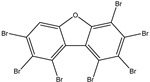	−9.8	−10.0					

^a^ Data sorted according to the expensive docking scores. ^b^ Taken from T3DB website ^25^. ^c^ Conv. stands for the conventional docking computation. ^d^ Exp. stands for the expensive docking estimation.

**Table 2:** Portended physiochemical parameters and structural descriptors of T3D2489, T3D2672, T3D2378, and XF7 as SARS-CoV-2 main protease (M^pro^) inhibitors.

Compound Name/Code	_mi_logP	TPSA	nON	nOHNH	Nrotb	MWt	%ABS
T3D2489	0.03	128.5	8	7	18	435.6	64.7%
T3D2672	6.7	163.7	13	4	9	842.1	52.5%
T3D2378	1.7	61.9	6	1	4	385.5	87.6%
XF7	4.3	109.9	8	2	6	492.5	71.1%

**Table 3:** Anticipated ADMET characteristics of the top potent inhibitors.

ADME Parameters	XF7	T3D2489	T3D2672	T3D2378
Absorption				
Water solubility	−3.5	−3.0	−3.4	−2.5
Caco2 permeability	0.5	−0.2	−0.1	0.3
Intestinal absorption (human)	93.5	47.6	56.7	95.0
Skin Permeability	−2.7	−2.7	−2.7	−3.2
P-glycoprotein substrate	Yes	Yes	Yes	Yes
P-glycoprotein I inhibitor	Yes	No	Yes	No
P-glycoprotein II inhibitor	Yes	No	Yes	No
Distribution				
VDss (human)	0.3	1.6	0.6	1.3
BBB permeability	−1.1	−0.7	−1.7	0.2
CNS permeability	−2.5	−4.1	−3.4	−2.6
Metabolism				
CYP2D6 substrate	No	No	No	No
CYP3A4 substrate	Yes	No	Yes	Yes
CYP1A2 inhibitior	No	No	No	No
CYP2C19 inhibitior	Yes	No	No	No
CYP2C9 inhibitior	Yes	No	No	No
CYP2D6 inhibitior	No	No	No	No
CYP3A4 inhibitior	Yes	No	No	No
Excretion				
Total Clearance	0.8	1.4	−0.1	0.8
Toxicity				
AMES toxicity	No	No	No	No
Max. tolerated dose (human)	0.4	0.3	−0.3	−0.4
hERG I inhibitor	No	No	No	No
hERG II inhibitor	Yes	Yes	No	Yes
Oral Rat Acute Toxicity (LD50)	3.3	2.7	2.8	2.4
Oral Rat Chronic Toxicity (LOAEL)	1.1	3.1	2.7	0.7
Hepatotoxicity	Yes	Yes	Yes	Yes
Skin Sensitisation	No	No	No	No
T. Pyriformis toxicity	0.3	0.3	0.3	0.6
Minnow toxicity	2.4	2.2	0.9	4.7

## Data Availability

The data presented in this study are available in the article and supplementary material.

## Supplementary Materials

The following supporting information can be downloaded at: https://www.mdpi.com/article/10.3390/ph15020153/s1, Figure S1: 2D representations of the binding modes of the thirty-two potent toxins complexed with SARS-CoV-2 main protease (M^pro^); Figure S2: Schematic representation of the utilized in silico techniques and the filtration process; Table S1: Estimated conventional and expansive docking scores (in kcal/mol), for top 200 toxins towards SARS-CoV-2 main protease (M^pro^); Table S2: Estimated conventional and expansive docking scores (in kcal/mol), and MM-GBSA binding energies (in kcal/mol) over 5 ns MD simulations for XF7 and the top 32 potent toxins towards SARS-CoV-2 main protease (M^pro^); Table S3: Top 20 enriched pathways influenced by philanthotoxin (T3D2489) targets resulted from PEA analysis.
