# Supplementary material for: Exploring Toxins for Hunting SARS-CoV-2 Main Protease Inhibitors: Molecular Docking, Molecular Dynamics, Pharmacokinetic Properties, and Reactome Study
Source: Pharmaceuticals (Basel). 2022 Jan 27;15(2):153. doi: 10.3390/ph15020153 (PMC8875976; doi:10.3390/ph15020153)
Supplement: Supplementary file 1 [file pharmaceuticals-15-00153-s001.zip › pharmaceuticals-1564879-supplementary-done.pdf]

pharmaceuticals

Exploring Toxins for hunting SARS-CoV-2 Main Protease Inhibitors: Molecular docking, molecular dynamics, pharmacokinetic properties, and Reactome study

Mahmoud A. A. Ibrahim [1,*], Alaa H. M. Abdelrahman [1], Laila A. Jaragh-Alhadad [2,3,*], Mohamed A. M. Atia [4], Othman R. Alzahrani [5], Muhammad Naeem Ahmed [6], Moustafa Sherief Moustafa [2], Mahmoud E. S. Soliman [7], Ahmed M. Shawky [8], Paul W. Paré [9], Mohamed-Elamir F. Hegazy [10] and Peter A. Sidhom [11]

1 Computational Chemistry Laboratory, Chemistry Department, Faculty of Science, Minia University, Minia 61519, Egypt; a.abdelrahman@compchem.net
2 Department of Chemistry, Faculty of Science, Kuwait University, Safat 13060, Kuwait; mostafa_msm@hotmail.com
3 Cardiovascular and Metabolic Sciences Department, Lerner Research Institute, Cleveland Clinic, Cleveland, OH 44195, USA
4 Molecular Genetics and Genome Mapping Laboratory, Genome Mapping Department, Agricultural Genetic Engineering Research Institute (AGERI), Agricultural Research Center (ARC), Giza 12619, Egypt; matia@ageri.sci.eg
5 Department of Biology, Faculty of Science, University of Tabuk, Tabuk 71491, Saudi Arabia; o-alzahrani@ut.edu.sa
6 Department of Chemistry, The University of Azad Jammu and Kashmir, Muzaffarabad 13100, Pakistan; drnaeem@ajku.edu.pk
7 Molecular Modelling and Drug Design Research Group, School of Health Sciences, University of KwaZulu-Natal, Westville, Durban 4000, South Africa; soliman@ukzn.ac.za
8 Science and Technology Unit (STU), Umm Al-Qura University, Makkah 21955, Saudi Arabia; amesmail@uqu.edu.sa
9 Department of Chemistry & Biochemistry, Texas Tech University, Lubbock, TX 79409, USA; paul.pare@ttu.edu
10 Chemistry of Medicinal Plants Department, National Research Centre, 33 El-Bohouth St., Dokki, Giza 12622, Egypt; elamir77@live.com
11 Department of Pharmaceutical Chemistry, Faculty of Pharmacy, Tanta University, Tanta 31527, Egypt; peter.ayoub@pharm.tanta.edu.eg

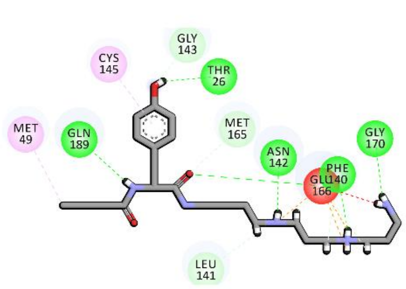

T3D2489

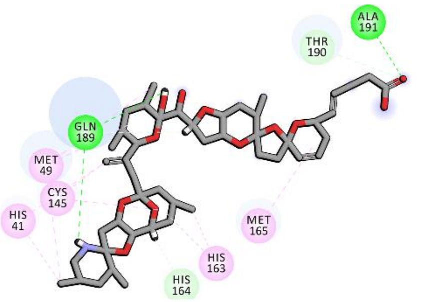

T3D2672

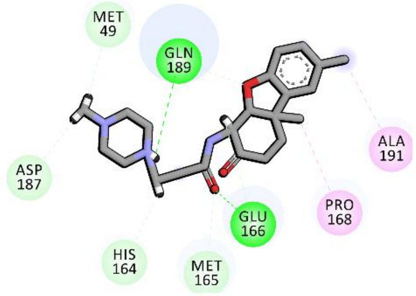

T3D2378

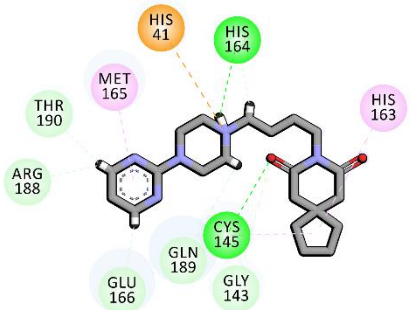

T3D2807

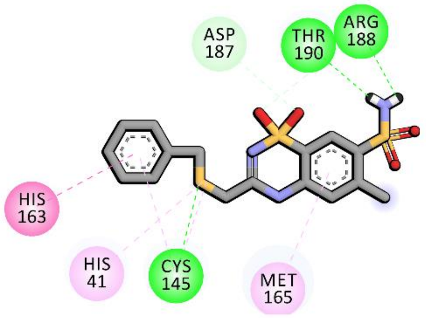

T3D2825

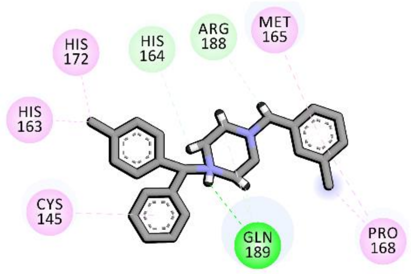

T3D2874

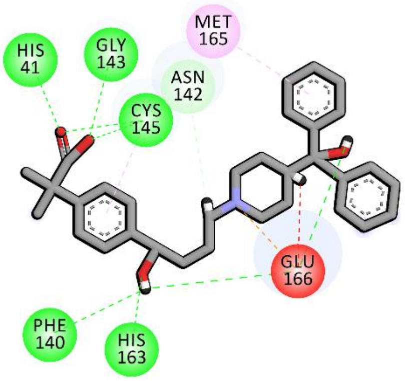

T3D2938

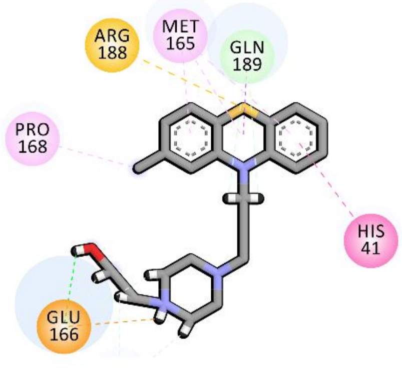

T3D2913

Figure S1. 2D representations of the binding modes of the thirty-two potent toxins complexed with SARS-CoV-2 main protease (M^{pro}).

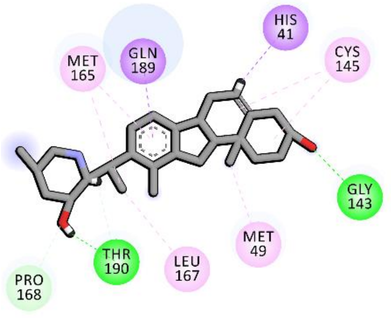

T3D4084

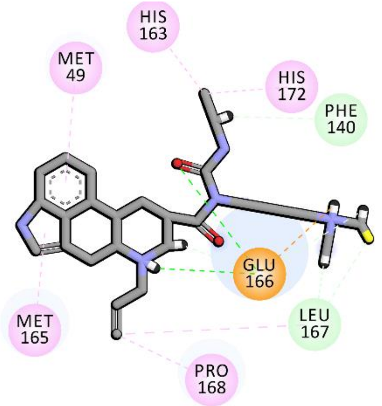

T3D2727

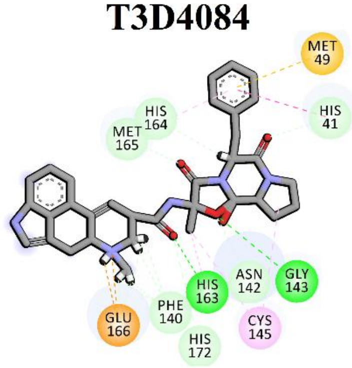

T3D2460

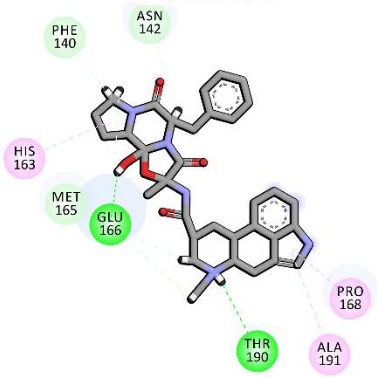

T3D2750

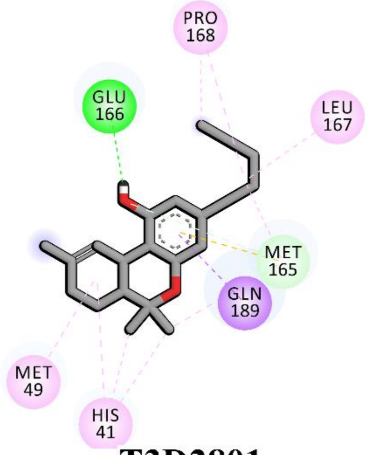

T3D2801

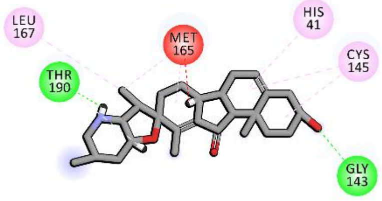

T3D4083

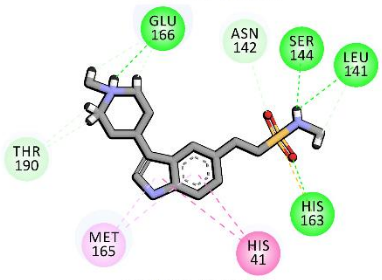

T3D2939

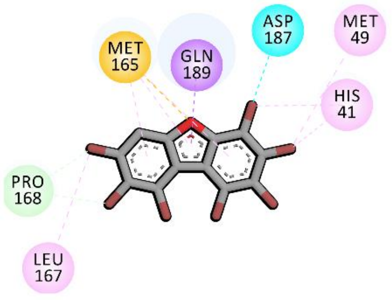

T3D2143

Figure S1. *Continued.*

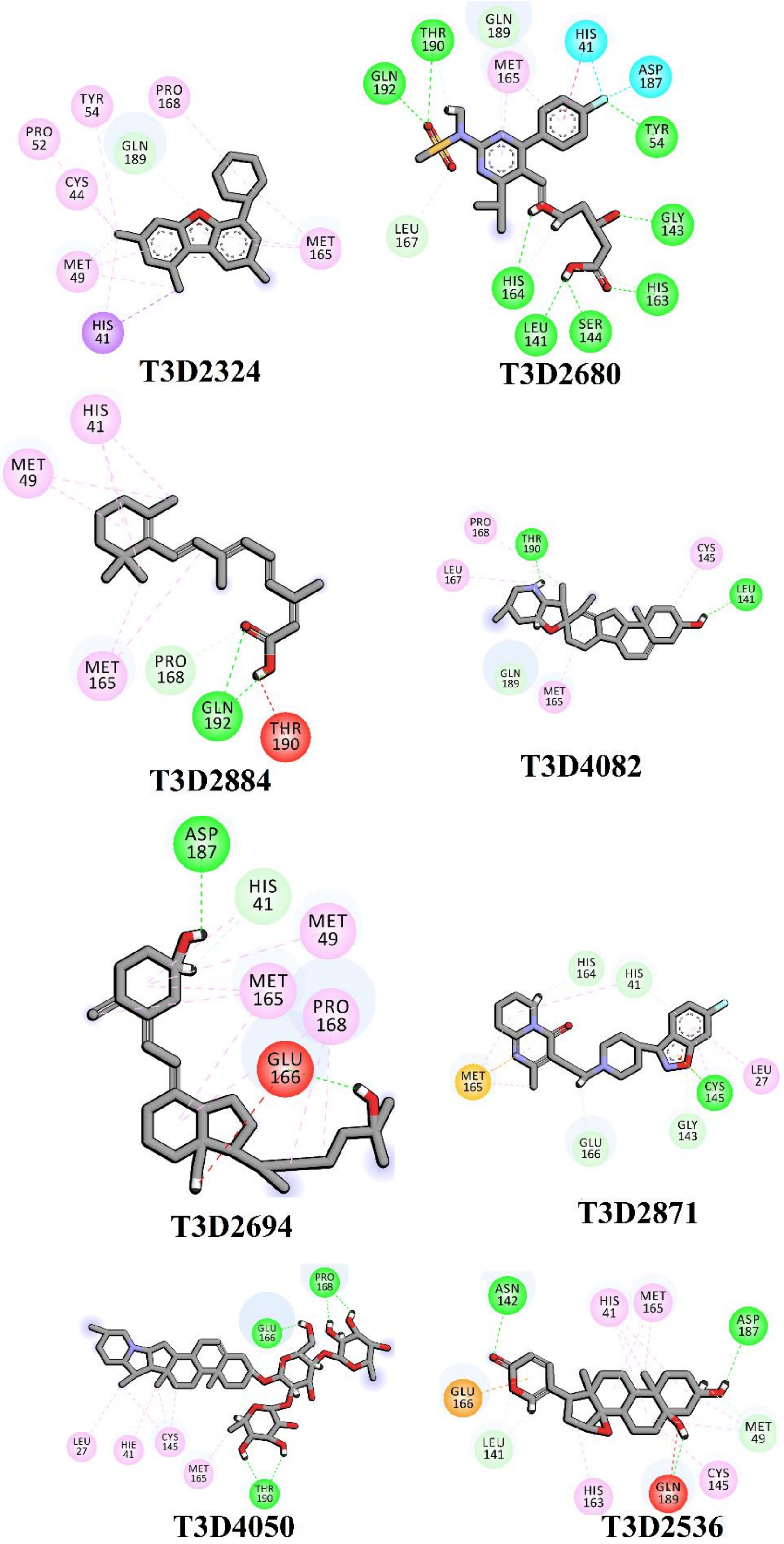

Figure S1. *Continued.*

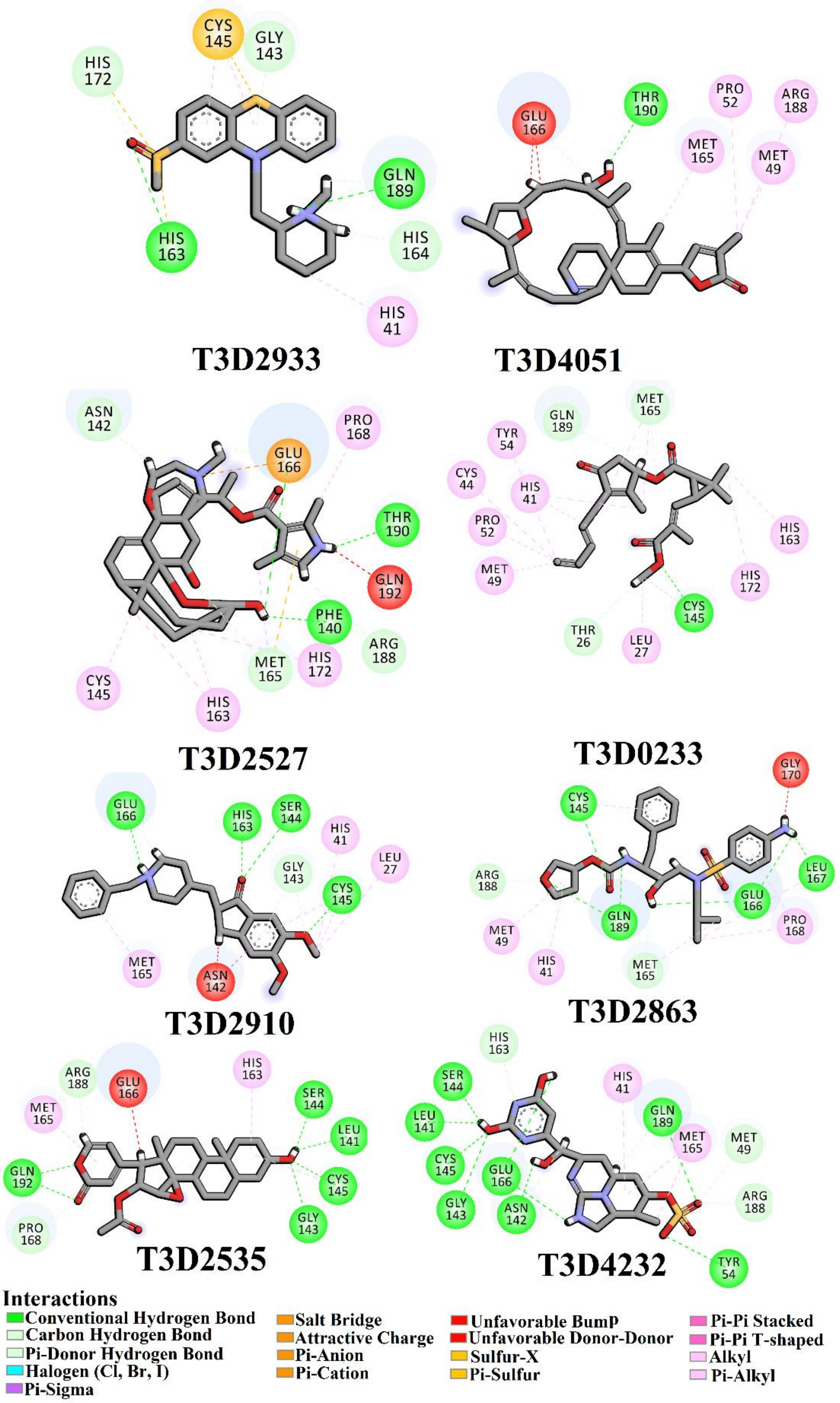

Figure S1. *Continued.*

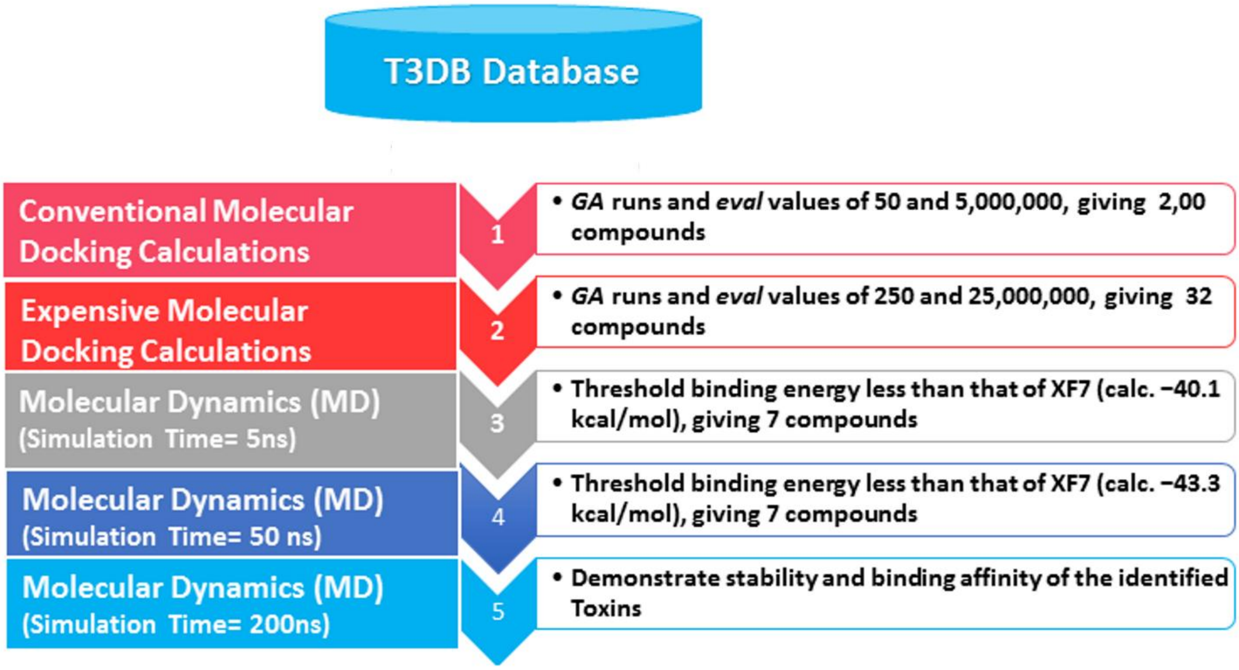

Figure S2. Schematic representation of the utilized *in silico* techniques and the filtration process.

Table S1. Estimated conventional and expansive docking scores (in kcal/mol), for top 200 toxins towards SARS-CoV-2 main protease (M^{pro}).[a]

No.	Compound Name/Code	Docking Score (kcal/mol)		No.	Compound Name/Code	Docking Score (kcal/mol)		No.	Compound Name/Code	Docking Score (kcal/mol)	
		Conv.[b]	Exp.[c]			Conv.[b]	Exp.[c]			Conv.[b]	Exp.[c]
	XF7	–9.1	–9.5								
1	T3D2489	–11.7	–11.7	54	T3D0015	–9.1	–9.1	107	T3D2528	–8.5	–8.6
2	T3D2672	–11.3	–11.6	55	T3D3925	–9.1	–9.1	108	T3D2755	–8.5	–8.5
3	T3D2378	–11.2	–11.2	56	T3D3887	–9.1	–9.1	109	T3D4088	–8.3	–8.5
4	T3D2807	–10.9	–10.9	57	T3D4512	–8.6	–9.1	110	T3D2985	–8.5	–8.5
5	T3D2825	–10.9	–10.9	58	T3D3726	–9.0	–9.0	111	T3D2753	–8.5	–8.5
6	T3D2874	–10.8	–10.9	59	T3D2162	–9.0	–9.0	112	T3D2796	–8.5	–8.5
7	T3D2938	–10.5	–10.8	60	T3D2483	–8.6	–9.0	113	T3D0636	–8.5	–8.5
8	T3D2913	–10.5	–10.8	61	T3D3742	–9.0	–9.0	114	T3D2044	–8.5	–8.5
9	T3D4084	–10.2	–10.6	62	T3D3752	–9.0	–9.0	115	T3D3907	–8.5	–8.5
10	T3D2727	–10.1	–10.5	63	T3D4957	–8.4	–9.0	116	T3D2951	–8.7	–8.5
11	T3D2460	–10.1	–10.2	64	T3D3722	–9.0	–9.0	117	T3D2015	–8.5	–8.5
12	T3D2750	–10.0	–10.2	65	T3D2532	–9.0	–9.0	118	T3D3746	–8.5	–8.5
13	T3D2801	–10.0	–10.1	66	T3D0629	–8.9	–8.9	119	T3D2004	–8.4	–8.4
14	T3D4083	–9.9	–10.1	67	T3D2757	–8.5	–8.9	120	T3D4235	–8.5	–8.4
15	T3D2939	–9.8	–10.0	68	T3D0174	–8.9	–8.9	121	T3D2836	–8.1	–8.4
16	T3D2143	–9.8	–10.0	69	T3D2978	–8.5	–8.9	122	T3D3782	–8.4	–8.4
17	T3D2324	–9.2	–9.9	70	T3D2892	–8.5	–8.9	123	T3D2677	–8.3	–8.4
18	T3D2680	–9.8	–9.9	71	T3D2043	–8.9	–8.9	124	T3D0010	–8.4	–8.4
19	T3D2884	–9.8	–9.9	72	T3D3721	–8.9	–8.9	125	T3D0062	–8.4	–8.4
20	T3D4082	–9.8	–9.8	73	T3D2995	–8.2	–8.8	126	T3D2002	–8.4	–8.4
21	T3D2694	–9.7	–9.8	74	T3D2358	–8.8	–8.8	127	T3D2837	–8.4	–8.4
22	T3D2871	–9.7	–9.8	75	T3D2697	–8.8	–8.8	128	T3D1672	–8.4	–8.4
23	T3D4050	–9.6	–9.8	76	T3D4032	–8.8	–8.8	129	T3D2988	–8.4	–8.4
24	T3D2536	–9.6	–9.8	77	T3D4343	–9.2	–8.8	130	T3D3781	–8.6	–8.4
25	T3D2933	–9.6	–9.7	78	T3D4910	–8.7	–8.8	131	T3D4056	–8.4	–8.4
26	T3D4051	–9.5	–9.7	79	T3D3762	–8.8	–8.8	132	T3D3834	–8.4	–8.4
27	T3D2527	–9.5	–9.7	80	T3D4314	–8.7	–8.8	133	T3D2134	–8.3	–8.3
28	T3D0233	–9.5	–9.7	81	T3D2700	–8.2	–8.8	134	T3D2328	–8.3	–8.3
29	T3D2910	–9.5	–9.6	82	T3D2017	–8.8	–8.8	135	T3D3914	–8.3	–8.3
30	T3D2863	–9.5	–9.6	83	T3D3891	–8.7	–8.8	136	T3D2773	–8.3	–8.3
31	T3D2535	–9.4	–9.6	84	T3D3712	–8.7	–8.7	137	T3D2338	–8.3	–8.3
32	T3D4232	–9.3	–9.6	85	T3D3729	–8.8	–8.7	138	T3D3915	–8.2	–8.3
33	T3D4068	–9.2	–9.5	86	T3D2975	–8.7	–8.7	139	T3D2001	–8.1	–8.3
34	T3D3767	–9.2	–9.5	87	T3D2984	–8.6	–8.7	140	T3D2911	–8.4	–8.3
35	T3D4988	–8.2	–9.5	88	T3D2045	–8.7	–8.7	141	T3D2012	–8.4	–8.3
36	T3D4923	–9.2	–9.5	89	T3D3847	–8.7	–8.7	142	T3D2751	–8.3	–8.3
37	T3D3778	–9.2	–9.5	90	T3D2041	–8.7	–8.7	143	T3D4283	–8.3	–8.3
38	T3D4916	–9.2	–9.5	91	T3D3880	–8.7	–8.7	144	T3D2137	–8.3	–8.3
39	T3D0631	–9.2	–9.5	92	T3D2968	–8.6	–8.7	145	T3D2040	–8.3	–8.3
40	T3D3733	–9.2	–9.5	93	T3D2920	–8.6	–8.7	146	T3D0548	–8.3	–8.3
41	T3D4242	–9.2	–9.5	94	T3D4937	–8.0	–8.7	147	T3D2188	–8.3	–8.3
42	T3D3732	–9.2	–9.5	95	T3D2703	–8.6	–8.7	148	T3D0632	–8.3	–8.3
43	T3D3763	–9.2	–9.5	96	T3D2008	–8.7	–8.7	149	T3D1673	–8.2	–8.3
44	T3D4963	–8.8	–9.5	97	T3D3710	–8.7	–8.7	150	T3D3768	–8.3	–8.3
45	T3D3777	–9.2	–9.5	98	T3D2858	–8.6	–8.7	151	T3D2026	–8.3	–8.3
46	T3D4914	–9.2	–9.5	99	T3D4961	–8.5	–8.6	152	T3D2722	–8.2	–8.3
47	T3D2907	–8.7	–9.5	100	T3D2692	–8.4	–8.6	153	T3D2003	–8.3	–8.3
48	T3D3779	–9.2	–9.5	101	T3D3875	–9.2	–8.6	154	T3D2897	–8.1	–8.3
49	T3D3709	–9.2	–9.5	102	T3D2852	–8.5	–8.6	155	T3D4087	–8.3	–8.3
50	T3D3748	–9.2	–9.5	103	T3D1147	–8.6	–8.6	156	T3D2145	–8.3	–8.3
51	T3D4322	–8.5	–9.5	104	T3D1649	–8.1	–8.6	157	T3D2699	–8.2	–8.3
52	T3D2961	–9.2	–9.4	105	T3D2997	–8.3	–8.6	158	T3D2789	–8.3	–8.3
53	T3D3734	–9.2	–9.3	106	T3D4240	–8.5	–8.6	159	T3D3744	–8.2	–8.2

Table S1. *Continued.*

No.	Compound Name/Code	Docking Score (kcal/mol)		No.	Compound Name/Code	Docking Score (kcal/mol)		No.	Compound Name/Code	Docking Score (kcal/mol)	
		Conv.[b]	Exp.[c]			Conv.[b]	Exp.[c]			Conv.[b]	Exp.[c]
160	T3D4021	–8.2	–8.2	173	T3D2007	–8.2	–8.2	187	T3D0516	–8.1	–8.1
161	T3D3747	–8.6	–8.2	174	T3D3720	–8.2	–8.2	188	T3D2154	–8.1	–8.1
162	T3D4245	–8.2	–8.2	175	T3D3838	–8.2	–8.2	189	T3D0578	–8.1	–8.0
162	T3D4504	–8.2	–8.2	176	T3D2158	–8.1	–8.1	190	T3D2147	–8.0	–8.0
163	T3D2742	–8.2	–8.2	177	T3D3800	–8.1	–8.1	191	T3D3780	–8.1	–8.0
164	T3D2888	–8.2	–8.2	178	T3D3745	–8.1	–8.1	192	T3D4086	–8.0	–8.0
165	T3D2917	–8.2	–8.2	179	T3D4103	–8.1	–8.1	193	T3D2139	–8.0	–8.0
166	T3D3759	–8.1	–8.2	180	T3D2929	–8.0	–8.1	194	T3D2673	–8.0	–8.0
167	T3D2886	–8.1	–8.2	181	T3D2469	–8.0	–8.1	195	T3D2966	–8.3	–8.0
168	T3D2115	–8.2	–8.2	182	T3D1221	–8.1	–8.1	196	T3D2011	–8.1	–8.0
169	T3D4092	–8.2	–8.2	183	T3D2842	–8.1	–8.1	197	T3D4942	–8.1	–8.0
170	T3D4983	–8.2	–8.2	184	T3D4064	–8.1	–8.1	198	T3D4029	–9.2	–8.0
171	T3D0622	–8.2	–8.2	185	T3D2101	–8.1	–8.1	199	T3D2670	–8.4	–8.0
172	T3D2010	–8.2	–8.2	186	T3D0495	–8.1	–8.1	200	T3D4030	–8.4	–8.0

[a]Data sorted according to the expensive docking scores.

[b]Conv. stands for the conventional docking calculation.

[c]exp. stands for the expensive docking calculation.

Table S2. Estimated conventional and expansive docking scores (in kcal/mol), and MM-GBSA binding energies (in kcal/mol) over 5 ns MD simulations for XF7 and the top 32 potent toxins towards SARS-CoV-2 main protease (M^{pro}).[a]

No.	Compound Name/Code	Docking Score (kcal/mol)		MM–GBSA Binding Energy (kcal/mol)
		Conv.[b]	Exp.[c]	
	XF7	−9.1	−9.5	−40.1
1	T3D2489	–11.7	–11.7	–54.7
2	T3D2672	–11.3	–11.6	–53.1
3	T3D2807	–10.9	–10.9	–48.2
4	T3D2378	–11.2	–11.2	–48.1
5	T3D2825	–10.9	–10.9	–42.8
6	T3D2938	–10.5	–10.8	–42.8
7	T3D2460	–10.1	–10.2	–42.0
8	T3D2874	–10.8	–10.9	–40.0
9	T3D2727	–10.1	–10.5	–39.9
10	T3D2750	–10.0	–10.2	–39.9
11	T3D2913	–10.5	–10.8	–39.9
12	T3D4084	–10.2	–10.6	–39.9

13	T3D4083	–9.9	–10.1	–36.4
14	T3D2801	–10.0	–10.1	–35.9
15	T3D2939	–9.8	–10.0	–33.7
16	T3D2143	–9.8	–10.0	–32.8
17	T3D2324	–9.2	–9.9	–32.5
18	T3D2680	–9.8	–9.9	–32.1
19	T3D2884	–9.8	–9.9	–31.6
20	T3D4082	–9.8	–9.8	–31.5
21	T3D2694	–9.7	–9.8	–31.1
22	T3D2871	–9.7	–9.8	–30.7
23	T3D4050	–9.6	–9.8	–30.6
24	T3D2536	–9.6	–9.8	–30.5
25	T3D2933	–9.6	–9.7	–29.1
26	T3D4051	–9.5	–9.7	–28.9
27	T3D2527	–9.5	–9.7	–27.8
28	T3D0233	–9.5	–9.7	–24.1
29	T3D2910	–9.5	–9.6	–23.7
30	T3D2863	–9.5	–9.6	–22.3
31	T3D2535	–9.4	–9.6	–21.2
32	T3D4232	–9.3	–9.6	–21.0

[a]Data sorted according to MM-GBSA binding energy over 5 ns MD Simulations.

[b]Conv. stands for the conventional docking calculation.

[c]exp. stands for the expensive docking calculation.

Table S3. Top 20 enriched pathways influenced by philanthotoxin (T3D2489) targets resulted from PEA analysis.

Pathway name	Entities				Reactions	
	found	ratio	pValue	FDR	found	ratio
Interleukin-4 and Interleukin-13 signaling	17 / 351	0.016	8.88E-16	8.46E-13	24 / 47	0.003
Activation of Matrix Metalloproteinases	9 / 74	0.003	5.73E-13	2.73E-10	22 / 27	0.002
Collagen degradation	9 / 79	0.004	1.02E-12	3.24E-10	17 / 34	0.003
Degradation of the extracellular matrix	11 / 224	0.01	2.07E-11	4.94E-09	60 / 105	0.008
Assembly of collagen fibrils and other multimeric structures	6 / 79	0.004	8.03E-08	1.53E-05	1 / 26	0.002
Interleukin-10 signaling	9 / 175	0.008	7.79E-06	1.23E-03	7 / 15	0.001
Collagen formation	6 / 207	0.01	2.00E-05	2.72E-03	1 / 77	0.006
Trafficking and processing of endosomal TLR	5 / 61	0.003	2.35E-05	2.80E-03	3 / 7	0.001
Extracellular matrix organization	13 / 1014	0.047	6.88E-05	7.22E-03	66 / 319	0.024
CLEC7A/inflammasome pathway	3 / 30	0.001	7.71E-05	7.32E-03	2 / 4	0
RUNX1 regulates transcription of genes involved in differentiation of keratinocytes	3 / 43	0.002	2.21E-04	1.68E-02	2 / 8	0.001
Signaling by Interleukins	26 / 2524	0.116	2.30E-04	1.68E-02	147 / 493	0.036

Extra-nuclear estrogen signaling	11 / 462	0.021	2.31E-04	1.68E-02	17 / 38	0.003
p75NTR negatively regulates cell cycle via SC1	2 / 9	0	2.80E-04	1.91E-02	1 / 3	0
CTLA4 inhibitory signaling	3 / 61	0.003	6.11E-04	3.85E-02	3 / 5	0
Negative regulation of the PI3K/AKT network	6 / 324	0.015	1.76E-03	1.04E-01	8 / 10	0.001
Interleukin-1 processing	2 / 25	0.001	2.10E-03	1.18E-01	2 / 5	0
MHC class II antigen presentation	5 / 235	0.011	3.67E-03	1.91E-01	4 / 26	0.002
Thrombin signalling through proteinase activated receptors (PARs)	5 / 121	0.006	4.26E-03	2.10E-01	9 / 15	0.001
Immune System	40 / 6071	0.28	4.51E-03	2.10E-01	272 / 1623	0.12
